# Scale‐free Spatio‐temporal Correlations in Conformational Fluctuations of Intrinsically Disordered Proteins

**DOI:** 10.1002/advs.202412989

**Published:** 2025-01-14

**Authors:** Haoyu Song, Jian Cui, Guorong Hu, Long Xiong, Yanee Wutthinitikornkit, Hai Lei, Jingyuan Li

**Affiliations:** ^1^ School of Physics Zhejiang University Hangzhou 310058 PR China; ^2^ Collaborative Innovation Center of Advanced Microstructures National Laboratory of Solid State Microstructure Department of Physics Nanjing University Nanjing 210093 PR China; ^3^ School of Physics and Astronomy Yunnan University Kunming 650091 PR China

**Keywords:** collective behavior, conformational dynamics, critical phenomena, intrinsically disordered proteins, scale‐free correlation

## Abstract

The self‐assembly of intrinsically disordered proteins (IDPs) into condensed phases and the formation of membrane‐less organelles (MLOs) can be considered as the phenomenon of collective behavior. The conformational dynamics of IDPs are essential for their interactions and the formation of a condensed phase. From a physical perspective, collective behavior and the emergence of phase are associated with long‐range correlations. Here the conformational dynamics of IDPs and the correlations therein are analyzed, using µs‐scale atomistic molecular dynamics (MD) simulations and single‐molecule Förster resonance energy transfer (smFRET) experiments. The existence of typical scale‐free spatio‐temporal correlations in IDP conformational fluctuations is demonstrated. Their conformational evolutions exhibit “1/*f* noise” power spectra and are accompanied by the appearance of residue domains following a power‐law size distribution. Additionally, the motions of residues present scale‐free behavioral correlation. These scale‐free correlations resemble those in physical systems near critical points, suggesting that IDPs are poised at a critical state. Therefore, IDPs can effectively respond to finite differences in sequence compositions and engender considerable structural heterogeneity which is beneficial for IDP interactions and phase formation.

## Graphical abstract

Intrinsically disordered proteins (IDPs), which have large conformational fluctuations, can collectively undergo liquid‐liquid phase separation. This study reveals that IDPs exhibit scale‐free spatiotemporal correlations in their conformation dynamics. It suggests that IDPs are poised at a critical state, providing useful insight into their emergent collective behavior.

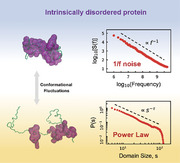

## 1 Introduction

Proteins and other biological macromolecules can self‐assemble into various membraneless organelles (MLOs), also known as biomolecular condensates.^[^ ^1^, ^2^ ^]^ This emergent collective behavior plays a pivotal role in a wide range of cellular processes and is implicated in numerous diseases.^[^ ^1^, ^3^, ^4^ ^]^ From a physical perspective, the collective behavior in living systems should be associated with long‐range correlations and the emergence of diverse phases.^[^ ^5^, ^6^ ^]^ The spontaneous phase separation and the formation of MLOs can be mediated by intrinsically disordered proteins (IDPs).^[^ ^1^, ^7^ ^]^ IDPs lack well‐defined native structures and continuously undergo large conformational fluctuations, which is apparently apart from structured proteins.^[^ ^8^, ^9^, ^10^ ^]^ Their inherent conformational dynamics are crucial for IDP interactions and the formation of the condensed phase.^[^ ^11^, ^12^ ^]^ In order to understand the mechanism of this biological emergent collective behavior and investigate the underlying long‐range correlations, a systematic study on the conformational dynamics of IDPs is highly demanded.

As revealed in various experimental studies, the conformational dynamics of IDPs are very complex, spanning multiple time and length scales.^[^ ^8^, ^11^ ^]^ This complexity is related to a multitude of coupled processes, including the local motions of individual residues, segmental motions of several residues, and conformational reconfiguration of the whole protein.^[^ ^9^, ^13^ ^]^ It should be noted that the living systems are generally multi‐scale and complex, wherein the dynamics at different time/length scales are often coherent and exhibit long‐range correlations.^[^ ^14^, ^15^ ^]^ Compared to the studies of collective behavior in macroscopic living systems such as neural networks and natural swarms,^[^ ^14^, ^16^ ^]^ the explorations in microscopic biological systems often face more challenges due to the resolution limitations of experimental techniques. Moreover, systematic studies about the spatiotemporal correlation in complex IDP systems are largely elusive. Interestingly, there have been some evidences suggesting the presence of correlations in the conformational dynamics of IDPs,^[^ ^17^, ^18^, ^19^ ^]^ such as the associations of conformational fluctuations among their distal residues.

In this paper, we systematically characterize the spatio‐temporal correlations in IDP conformational dynamics. Three IDPs that are important for the formation of MLOs are considered. The conformational evolutions of IDPs are investigated with µs‐scale all‐atom molecular dynamics (MD) simulations. Dynamic “residue domains”, formed by reversible assemblies of residues, are observed during the conformational fluctuations of all these IDPs. The sizes of the residue domains follow power‐law distributions, meanwhile, the power spectra of the global conformational fluctuations exhibit “1/*f* noise”. These reflect the existence of scale‐free spatial and temporal correlations in IDP conformational dynamics, resembling other physical and biological systems near critical points.^[^ ^14^, ^16^, ^20^, ^21^ ^]^ Being in a critical state allows these IDPs to effectively respond to the finite differences of residue compositions along their sequences, representing heterogeneous structural heterogeneity. This is beneficial for the IDP interactions and their collective behavior in the emergence of the condensed phase.

## 2 Results and Discussion

### 2.1 The Power Spectrum of the Global Conformational Fluctuations Exhibits “1/***f*** Noise”

**Figure 1:**
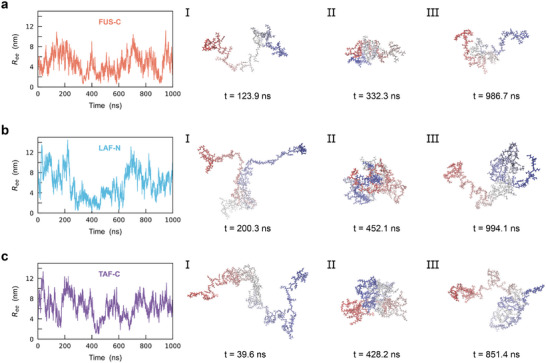
Large‐scale conformational fluctuations of three typical intrinsically disordered proteins (IDPs). a) Left: The end‐to‐end distance (*R_ee_*) of FUS‐C. The trajectory shown is one of the ten 1‐µs trajectories. Right: Representative conformations of FUS‐C at different points in the trajectory. The N‐ to C‐ termini are colored from red to blue. Same as in a) for b) LAF‐N and c) TAF‐C.

We investigate the conformational dynamics of three IDPs (Figure S1, Supporting Information): the C‐terminal domain of FUS (FUS‐C; 73 residues),^[^ ^22^ ^]^ the N‐terminal domain of LAF‐1 (LAF‐N; 168 residues)^[^ ^23^ ^]^ and the C‐terminal region of TAF15 (TAF‐C; 207 residues),^[^ ^24^ ^]^ respectively. They are all important for the formation of MLOs and are classified as RGG‐rich motifs.^[^ ^25^, ^26^, ^27^ ^]^ The conformational dynamics of the IDPs are depicted based on 10 independent 1‐µs simulations for each protein. **Figure** **1** shows the end‐to‐end distance (*R_ee_*) and the conformations along a representative trajectory. These proteins continuously undergo large‐scale conformational fluctuations. The mean values of *R_ee_* of three IDPs are 5.12 ± 2.01 nm (FUS‐C), 6.83 ± 3.07 nm (LAF‐N) and 6.00 ± 2.54 nm (TAF‐C; Figure S2, Supporting Information). Our *R_ee_* distribution of LAF‐N is similar to that in previous study.^[^ ^12^ ^]^

**Figure 2:**
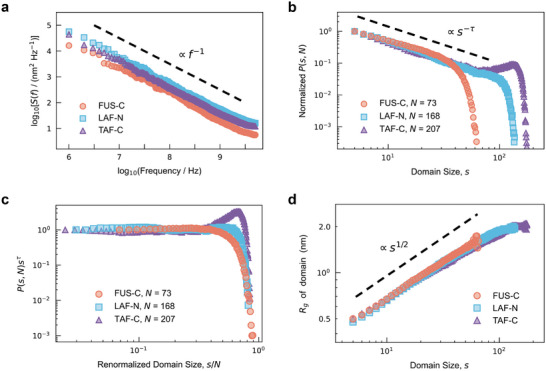
The 1/***f*** behavior in IDP conformational dynamics and scale‐invariant residue domains. a) The power spectrum *S*(*f*) of the evolution of the end‐to‐end distance. The power spectra of FUS‐C (orange), LAF‐N (blue), and TAF‐C (purple) all show power law forms for more than three frequency orders of magnitude, and they share similar exponents (≈‐1). The dashed reference line is plotted as a visual guide. b) The size distributions of the residue domains. The domain size *s* is the number of residues contained in each domain. The maximum of the distribution is normalized to one. The size distributions follow power law forms. The exponent of the dashed reference line τ is 1.15. The deviation from the power law in the tail of the distribution is largely attributed to the finite size effect. c) Finite‐size scaling of the domain size distributions. The domain size *s* is renormalized based on the IDP system size *N*, i.e., the sequence length. d) Radius of gyration of the domain versus the domain size, illustrating the fractal domain structure.

To characterize the conformational dynamics of these IDPs, the power spectra *S*(*f*) of their end‐to‐end distance *R_ee_* (*t*) are then calculated (**Figure** **2**a) by Fourier transforming the autocorrelation function of *R_ee_* (*t*) as follows:^[^ ^20^ ^]^

(1) $$S\left(f\right)=\int \langle R_{ee}\left(t_{0}+t\right)R_{ee}\left(t_{0}\right)\rangle e^{-2\pi ift}dt$$

where the angular brackets 〈 · · · 〉 represent an average overall times *t*_0_, and the power spectrum is calculated as the average of all 10 independent trajectories. These IDPs do not show characteristic frequencies. Strikingly, their power spectra all scale with the power law *S*(*f*)∝*f* ^−β^ for more than three orders of magnitude. The power‐law power spectrum ensures the self‐similarity of dynamics in the sense that its behavior remains the same on whatever scale we look at it, which is also called scale‐free property.^[^ ^28^ ^]^ Thus, the conformational dynamics of all these IDPs are self‐similar.^[^ ^29^, ^30^ ^]^ Moreover, the exponents β of three IDPs are all close to 1, approximately following *S*(*f*)∝*f* ^−1^. This kind of scale‐free power spectrum is often referred to as “1/*f* noise” and exhibits long‐range temporal correlation,^[^ ^31^, ^32^ ^]^ as an intermediate between white noise (β = 0) and Brownian noise (β = 2). Therefore, the 1/*f* behavior demonstrates the scale‐free temporal correlations in the global conformational fluctuations of these IDPs.

In addition, we notice polymers near the θ‐point can also display large conformational fluctuations. We consider the conformational dynamics of such polymer to better understand the long‐range temporal correlation in IDPs. The polymers are composed of polar and hydrophobic beads. In each polymer sequence, the proportion of hydrophobic beads is ≈0.5, while their positions are settled randomly (Figure S4, Supporting Information). A total of 100 independent polymer sequences are constructed. These polymers exhibit a similar global dimension to IDP: Their *R_ee_* distribution and Flory exponent are almost identical to those of IDP (Figure S5a, Supporting Information). Notably, the conformational dynamics of these polymers are essentially different from IDP. The *S*(*f*) of polymers drops out of the 1/*f* behavior in the low‐frequency region (Figure S5e, Supporting Information). The exponent β in this region is merely ≈0.54, significantly biased towards white noise. It suggests that even though the polymer system shares similar large conformational fluctuations and Flory exponent with IDP, its conformational dynamics fail to present “1/*f* noise” and scale‐free temporal correlation.

### 2.2 Power‐Law Size Distribution of Residue Domains and Finite‐Size Scaling

We notice that the conformational fluctuations of IDPs can be attributed to the evolution of dynamic “residue domains”, i.e., assemblies of residues that spontaneously form and dissolve. The size of these domains varies greatly, ranging from small domains composed of a few residues to large domains containing more than one‐third of the entire sequence. Such dynamic residue domains were also observed in previous single‐molecule experiments.^[^ ^33^, ^34^, ^35^ ^]^

The distributions of the domain size *s* (the number of residues contained in each domain) are investigated for three IDPs (Figure 2b). The distributions are broad and lack characteristic size. All three distributions follow the power law *P*(*s*) ~ *s* ^−τ^ with similar slopes (exponent τ ≈ 1.15). The deviation from the power law in the tail of the distribution can be attributed to the finite size effect.^[^ ^36^ ^]^ The domain size *s* is restricted by the system size (i.e., the protein size *N*, namely, the number of residues in the sequence). Consequently, the size distribution of the residue domain can be described with the consideration of the protein size *N* as follows:

(2) $$P\left(s,N\right)∼s^{-\tau }G\left(\frac{s}{N}\right)$$

The power law *s* ^−τ^ is modified by a dimensionless scaling function G,^[^ ^5^, ^36^ ^]^ and the rescaled *P*(*s*, *N*)*s* ^τ^ versus *s*/*N* is shown in Figure 2c. After rescaling with protein size *N*, the distributions of all three proteins converge to a single curve. This collapse suggests that these IDPs share a similar scale‐free spatial correlation, which is often associated with scale‐invariant (fractal) structures.^[^ ^20^, ^37^ ^]^ Thus, the relationship between the domain size *s* and their radius of gyration is further discussed (Figure 2d). The radius of gyration scales with the domain size as a power law, $R_{g}∼s^{1/d_{f}}$ (*d_f_* ≈ 2). This indicates that the residue domains have fractal shapes with a dimension of ≈2.^[^ ^38^, ^39^ ^]^ In a word, IDP conformational fluctuations engender scale‐invariant domain structures with power‐law size distributions. The domain distribution of polymers is also calculated (Figure S5f, Supporting Information). It shows no constant slope and rapidly decreases with the increasing domain size. The distribution significantly deviates from power law, suggesting the absence of scale‐free spatial correlation in polymer systems.

Our analysis results of the conformational dynamics of IDPs, i.e., the power law behaviors in both the power spectra and domain size distributions, suggest that both temporal and spatial correlations are scale‐free. Both features are absent in the polymer system. Remarkably, the phenomena of 1/*f* noise and scale‐invariant domain structures are often regarded as spatiotemporal signatures of criticality.^[^ ^14^, ^20^, ^37^ ^]^ Thus, these findings collectively provide evidence that IDP conformational dynamics are near the critical state. This may be attributed to the self‐organized process of a dynamic system.^[^ ^20^, ^40^ ^]^

### 2.3 Scale‐Free Behavioral Correlation Between Relative Motions of Residues

**Figure 3:**
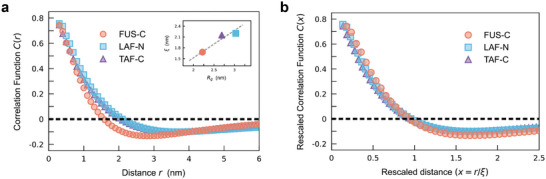
Correlations between the relative motions of the residues. a) The correlation function *C*(*r*) is the average inner product of the relative motions of residue pairs at mutual distance *r*. The correlation length ξ is defined by the zero crossing, indicated by the black dashed line. The ξ values for the three IDPs are 1.68 nm (FUS‐C; orange), 2.20 nm (LAF‐N; blue), and 2.15 nm (TAF‐C; purple). The inset shows their correlation lengths are ≈75% of the mean *R_g_*. b) Correlation function *C*(*x*) with respect to the rescaled variable *x* = *r*/ξ.

To comprehensively study the critical phenomena in IDP conformational dynamics, we also investigate the behavioral correlations between the motions of residues.^[^ ^16^, ^41^ ^]^ Specifically, the relative motion of residue ${\vec{u}}_{i}$ is considered by removing the translational and rotational motion of the whole protein. Then, the correlation between the relative motions of residue pairs $C_{ij}={\vec{u}}_{i}\;\cdot {\vec{u}}_{j}$ is calculated. The resulting correlation function *C*(*r*) is defined as follows:

(3) $$C\left(r\right)=\frac{\sum_{i<j}^{N}\frac{C_{ij}}{\sqrt{C_{ii}C_{jj}}}\delta \left(r-r_{ij}\right)}{\sum_{i<j}^{N}\delta \left(r-r_{ij}\right)}$$

where δ(*r* − *r_ij_*) is the Dirac delta function selecting residue pairs at mutual distance *r*. The correlation functions for three IDPs are shown in **Figure** **3**a. As *r* increases, the correlation decays from its maximum crosses zero, and reaches a negative minimum. The correlation length ξ is defined by the zero crossing. The correlation lengths of the three IDPs are 1.68 nm (FUS‐C), 2.20 nm (LAF‐N), and 2.15 nm (TAF‐C). They are close to the magnitudes of the whole proteins which can be depicted by their mean *R_g_* values (Figure S2, Supporting Information). The ratio of ξ to *R_g_* reaches up to 0.75. The rescaled correlation functions *C*(*x*) with respect to the rescaled variable *x* = *r*/ξ show a collapse (Figure 3b). Such scale‐free behavioral correlation is also consistent with the collective behaviors in a variety of biological and physical systems around the vicinity of critical points.^[^ ^16^, ^42^, ^43^ ^]^

### 2.4 Sequence‐Dependent Structural Heterogeneity

It has been well acknowledged that critical systems exhibit a duality of robustness and susceptibility.^[^ ^14^, ^44^ ^]^ Similar characteristics are also observed in IDP systems. For instance, IDPs can reliably perform their biological functions, meanwhile sensitively responding to diverse environmental stimuli.^[^ ^3^, ^45^, ^46^ ^]^ Being in the critical state may also be related to the intriguing response to the sequence composition.^[^ ^3^, ^47^, ^48^, ^49^, ^50^ ^]^ IDP sequences display high mutational tolerance and fast evolutionary rate, and many mutations barely influence the conformation behaviors of IDPs.^[^ ^3^, ^47^ ^]^ On the other hand, IDPs are sensitive to mutations at specific sites, which can change their conformational dynamics and lead to aggregation and diseases.^[^ ^48^, ^49^, ^50^ ^]^ Single mutations in some IDPs can induce substantial conformational changes,^[^ ^49^, ^50^ ^]^ e.g., the formation of aggregation‐prone β‐sheets. In addition, the difference in the residue composition along the sequence can lead to structural heterogeneity, e.g., local compactness, which is considered to be essential for IDPs to perform their biological functions.^[^ ^7^, ^51^, ^52^ ^]^

**Figure 4:**
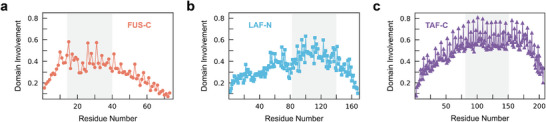
Structural heterogeneity along the IDP sequence. The domain involvement of the residues along the sequence of a) FUS‐C, b) LAF‐N, and c) TAF‐C. The domain‐prone regions of the sequence are identified as residues 15–39 in FUS‐C, 83–138 in LAF‐N, and 82–150 in TAF‐C (gray‐shaded areas).

We then investigate the sequence‐dependent structural heterogeneity in the three IDPs. As shown in the representative IDP conformations (Figure 1), the structural compactness is attributed to the residue domain, especially the dominant residue domain (the largest domain in each conformation). The tendency of residues involved in the dominant domain is shown in **Figure** **4**. All three IDPs exhibit considerable structural heterogeneity, with varying tendencies of domain involvement. The domain‐prone region in each IDP, i.e., the top third of the sequence, is identified: residues 15–39 in FUS‐C, 83–138 in LAF‐N and 82–150 in TAF‐C. Interestingly, even though the domain‐prone regions of the three IDPs are located at different positions (at the N‐terminal region in FUS‐C, whereas at the C‐terminal region in LAF‐N and TAF‐C), they share similar characteristics of residue compositions. Gly, Arg, and Tyr are modestly enriched in the domain‐prone regions of all three IDPs (Table S1, Supporting Information). It should be noted that these residues are thought to facilitate intra‐protein interactions: positively charged Arg and aromatic Tyr can mediate various types of interactions,^[^ ^53^, ^54^ ^]^ including hydrogen bonds, *sp* ^2^‐π interactions, cation‐π interactions, and Gly can enhance structural flexibility.^[^ ^55^, ^56^ ^]^ In short, all these IDPs share similar responses to the difference in residue compositions and manifest reliable structural heterogeneity.

### 2.5 Influence of Environment on the Critical State of IDP

We also study the behavior of IDP and the robustness of the critical state in varying environments. The conformational fluctuations of IDP in the solution with increased salt concentration are studied. It has been reported that the increase of salt concentration can substantially impact the conformation of IDP and the formation of a condensed phase.^[^ ^12^, ^57^ ^]^ Our simulation of FUS‐C in 500 mM NaCl solution shows that the protein becomes more compact (Figure S6a, Supporting Information). Notably, the population of large residue domain becomes dominant and the size distribution deviates significantly from the power law (Figure S6b, Supporting Information). In addition, the power spectrum shifts away from “1/*f* noise” and exhibits a tendency towards Brownian noise (Figure S6c, Supporting Information). These deviations may arise from the enhanced residue interactions such as cation‐π interactions with the increasing salt concentration.^[^ ^58^ ^]^ The loss of both spatial and temporal correlations indicates the deviation of the critical state of IDP at high‐salt conditions. More strikingly, the corresponding structural heterogeneity of FUS‐C is also diminished (Figure S6d, Supporting Information). The difference in domain involvement between N‐ and C‐ regions is largely reduced. Taken together, with the increase of environmental salt concentration, IDP deviates from the critical state, and the resulting structural heterogeneity is also eliminated.

### 2.6 Single‐Molecule FRET Experiments on Conformational Dynamics of IDPs

**Figure 5:**
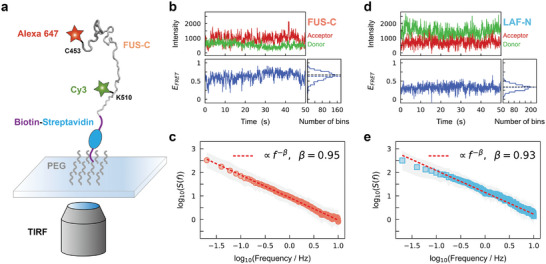
Single‐molecule Förster resonance energy transfer experiments confirm the temporal signature of criticality. a) Schematic diagram of the smFRET experiment. IDPs are immobilized on a PEG‐coated glass surface via a biotin‐streptavidin linkage and are labeled with the FRET dye pair Cy3‐Alexa‐647. Total internal reflection fluorescence (TIRF) microscopy is employed to monitor the fluorescence intensity. b) Representative fluorescence and FRET efficiency trajectories as well as the corresponding FRET efficiency histogram for FUS‐C. The mean FRET efficiency values 〈*E*〉 (blue dashed line) and 〈*E*〉_*simulation*_ (black dashed line; calculated from simulation results) are also shown in the histogram. c) The estimated power spectrum of FUS‐C (gray area for error bar: mean ± s.d., based on 62 trajectories). d) Representative fluorescence and FRET trajectories with corresponding efficiency histogram and (e) estimated power spectrum (error bar: mean ± s.d., based on 40 trajectories) for LAF‐N.

Our simulation results reveal that the conformational fluctuations of IDPs exhibit 1/*f* behavior, and such self‐similar dynamics serve as the temporal signature of the criticality of IDPs. To validate this finding, single‐molecule Förster resonance energy transfer (smFRET) experiments which can provide information on the distance between labeled residues are employed to probe the conformational dynamics of IDPs.^[^ ^59^, ^60^ ^]^ Picomolar concentrations of FUS‐C labeled with Cy3 as a FRET donor (at position K510) and Alexa 647 as an acceptor (at position C453) are immobilized on a polyethylene glycol (PEG)‐coated glass surface to perform the FRET experiments (**Figure** **5**a). Previous work indicates the effect of immobilization on conformational dynamics of IDP is very limited.^[^ ^61^ ^]^ The representative fluorescence and FRET efficiency trajectories of FUS‐C are shown in Figure 5b. The transfer efficiency trajectory exhibits considerable fluctuations, and the corresponding histogram has a single broad peak, which is consistent with previous experiments with other IDPs.^[^ ^61^, ^62^ ^]^ The mean FRET efficiency value 〈*E*〉 of ≈0.63 (blue dashed line in the histogram) matches well with the simulation value (〈*E*〉_*simulation*_ ≈ 0.67, black dashed line; Figure S7, Supporting Information). Subsequently, we estimate the distance between the FRET donor and acceptor based on the FRET efficiency trajectories and then calculate the power spectrum of the conformational fluctuations for FUS‐C (Figure 5c). The power spectrum follows the power law *S*(*f*)∝*f* ^−β^, with an exponent β = 0.95 (determined by a linear fit, *r* ^2^ > 0.99). The experimental result of exponent β is consistent with the simulation result, confirming the 1/*f* noise in the conformational fluctuations of FUS‐C. To investigate whether the labeling position would influence the results, we purify FUS‐C and relabel it at both termini (Figure S1, Supporting Information). The alternation shows negligible impact on the scaling of the power spectrum (Figure S8, Supporting Information). Additionally, LAF‐N is also investigated with smFRET experiments (Figure 5d). The 1/*f* behavior is observed in this IDP as well (Figure 5e). Taken together, both single‐molecule spectroscopic experiments and simulations observe the power‐law spectrum in IDPs with similar exponent. It indicates the same self‐similar dynamic behavior of IDPs in different timescales and verifies the 1/*f* behavior of the IDP conformational dynamics, a temporal signature of criticality. Moreover, the consistency of our simulation and experimental results suggests that the self‐similarity of IDP conformational dynamics is valid across extensive time scales, spanning from nanoseconds to seconds.

## 3 Conclusion 

In this work, we conduct a systematic analysis of the large‐scale conformational fluctuations of IDPs and reveal the typical scale‐free spatio‐temporal correlations which are the characteristics of critical systems.^[^ ^14^, ^20^, ^37^ ^]^ The critical phenomena in the conformational dynamics of the IDPs include 1/*f* noise, scale‐invariant domain structures and collective motion of residues. Several biological systems have been considered to live in the vicinity of criticality, e.g., the brain and natural swarms,^[^ ^14^, ^16^ ^]^ while these systems are predominantly at macroscopic scales. The study of microscopic biosystems is still challenging, largely due to the scarcity of experimental techniques. Our simulations combined with single‐molecule experiments clearly elucidate a molecular‐scale biological system poised at a critical state.

Being in the critical state allows IDPs to reliably perform their biological functions. In particular, criticality enables IDPs to effectively and consistently respond to their sequence features, and further engenders robust patterns of structural heterogeneity, facilitating the interactions of these proteins. For instance, LAF‐N has its N‐ and C‐termini more extended, both of which have been reported to be prone to the interactions between LAF‐N and crucial for its phase separation.^[^ ^53^, ^63^ ^]^ Besides, FUS‐C exhibits considerable heterogeneity between its N‐ and C‐regions, which preferentially bind to RNA and nuclear import receptor separately.^[^ ^64^, ^65^ ^]^ On the other hand, the dysfunction of IDPs in varying environments may be related to the failure of criticality and the reduction of structural heterogeneity. For example, high‐salt conditions weaken the nuclear import of FUS and result in its mislocalization into the cytoplasm.^[^ ^66^ ^]^ Our findings about the criticality of IDPs and the related structural heterogeneity provide useful insights into the mechanism of how IDPs reliably accomplish phase separation and other biological functions.

IDPs are crucial biomolecules mediating the formation of membraneless organelles (MLOs).^[^ ^1^, ^7^ ^]^ Apart from the usual thermodynamic perspective for investigating MLOs, such as characterizing interactions between IDPs,^[^ ^25^, ^53^ ^]^ MLOs can also be viewed as dynamic self‐organizing structures and the collective behavior of biomolecules in cells.^[^ ^67^, ^68^ ^]^ We study the dynamic properties of IDPs and find IDPs exhibit 1/*f* dynamics. We further perform power spectrum analysis on a condensate system consisting of 40 LAF‐N motifs (Figure S9 Supporting Information; trajectories from Ref.[69]). In this system, LAF‐N still displays 1/*f* dynamics, suggesting that IDPs may retain this property in condensates. The 1/*f* dynamics indicates that the motions of IDPs in different frequency decades,^[^ ^30^ ^]^ both large‐scale conformational fluctuations and the rapid rearrangement of local residue contacts, have comparable contributions. Previous studies have shown that both motions are important to MLOs. The large‐scale conformational fluctuations of IDPs promote the formation of condensates,^[^ ^12^ ^]^ while the rapid rearrangement of contacts is beneficial to maintain fluidity and molecular exchange of condensates.^[^ ^11^ ^]^ Our work contributes to further understanding of IDP dynamic behavior and its role in the MLOs.

It is worth noting that IDPs are ubiquitous in nature, with their proportion increasing from bacteria to higher organisms.^[^ ^70^ ^]^ Particularly, the phase‐separated MLOs mediated by IDPs play a central part in numerous cellular processes, such as stress response and RNA metabolism, and are associated with many human diseases, including neurodegenerative diseases and cancers.^[^ ^1^, ^4^, ^7^ ^]^ The present study can be extended to many other IDPs involved in phase separation. Systematic studies on the criticality of IDPs and the related structural heterogeneity would be helpful to better understand the physical nature underlying their collective behavior, especially the emergence of MLOs.

## 4 Experimental Section

### Computational Details

Three typical intrinsically disordered proteins (IDPs), i.e., FUS‐C (residues 454–526 of FUS), LAF‐N (residues 1–168 of LAF‐1) and TAF‐C (residues 386–592 of TAF15), were investigated using all‐atom molecular dynamics (MD) simulations. Their amino acid sequences are shown in Figure S1 (Supporting Information). For convenience, the residue indices of FUS‐C and TAF‐C were renumbered (i.e., residues 1–73 for FUS‐C and 1–207 for TAF‐C) in the main text. They were enriched with charged residues and were denoted as RGG‐rich motifs. The peptide chains of three IDPs were constructed by Pymol, followed by relaxation simulations of 100 ps in vacuum and 10 ns in water solution. The resulting conformation of each protein was then placed in a cubic box. The side length of the box was 12 nm (FUS‐C), 16 nm (LAF‐N), and 18 nm (TAF‐C), respectively. The boxes were solvated by water molecules, and sodium and chloride ions were added to neutralize the system and mimic physiological conditions (150 mM NaCl). Three systems all perform a 50‐ns simulation at a high temperature (450 K), from which 10 conformations were randomly selected for each protein. These conformations serve as the initial conformations for the subsequent production simulations: 10 independent 1000‐ns production runs (a total of 10 µs) for each protein at 300K.

The a99SB‐disp force field and TIP4P‐DE water model were used. The MD simulations were carried out using GROMACS. The production simulations were performed at the temperature of 300 K and the pressure of 1 bar, which were maintained using a velocity‐rescaled Berendsen thermostat and Parrinello–Rahman barostat, respectively. The periodic boundary conditions (PBC) were applied in all directions. Both the Van der Waals (VdW) interactions and short‐range electrostatic interactions were calculated within a cut‐off distance of 1.2 nm. Beyond this distance, long‐range electrostatic interactions were calculated using the Particle Mesh Ewald (PME) method. The LINCS algorithm was adopted to constrain the vibrations of covalent bonds with hydrogen atoms. The coordinates of systems were saved every 100 ps.

A coarse‐grained HP model with one bead per residue was used for the simulation of the polymers. Each polymer sequence was comprised of two types of beads, namely hydrophobic (H) and polar (P) beads. A harmonic potential was used to model interactions between bonded beads, and a modified Lennard–Jones potential was used to model nonbonded interactions.^[^ ^71^ ^]^ The proportion of hydrophobic residues was ≈0.5 for FUS‐C based on the Engelman GES Hydrophobicity Scale.^[^ ^72^, ^73^ ^]^ Accordingly, polymer sequences that had the same length as FUS‐C and half of hydrophobic beads were constructed. The positions of hydrophobic beads were randomly settled in each sequence, and a total of 100 independent sequences were generated (Figure S4, Supporting Information).

### The Calculation of the Power Spectrum

The temporal correlation of the conformational fluctuation of IDP was based on the analysis of the evolution of its global conformation, i.e., the distance between the residues in two termini. The temporal correlation could be reflected according to the shape of its power spectrum. The evolution of the residue distance is denoted as *R*(*t*). In simulations, *R*(*t*) is computed as the center‐of‐mass distance. In FRET experiments, *R*(*t*) is obtained from the relationship between transfer efficiency *E*(*t*) and distance as follows,

(4) $$E\left(t\right)=\frac{1}{1+{\left[\frac{R\left(t\right)}{R_{0}}\right]}^{6}}$$

where *R*_0_ is the spectroscopically determined Förster radius (*R*_0_ ≈ 5 nm for the FRET dye pair used in the experiments). The power spectrum *S*(*f*) is then calculated by Fourier transforming the autocorrelation function of *R*(*t*),

(5) $$S\left(f\right)=\int \langle R\left(t_{0}+t\right)R\left(t_{0}\right)\rangle e^{-2\pi ift}dt$$

where the angular brackets 〈 · · · 〉 represent an average overall times *t*_0_. For both simulations and experiments, the final power spectrum is calculated as the average of all independent trajectories.

### The Definition of Residue Domain

Residue domains represent the clusters of residues that were interconnected through contacts. A residue contact was defined when the minimum distance between any atom of two residues was less than 0.35 nm. The contacts of neighboring residues along the sequence, i.e., the contacts between residue i and i±1, were excluded. After the determination of contacts, the contact map of every conformation could be obtained. To quantitatively characterize the residue domains from the contact map, the k‐core decomposition was used. K‐core decomposition was a graph analysis algorithm used to find the connected subgraph of a network, in which each node had at least k neighbors in the subgraph.^[^ ^74^ ^]^ The connected subgraph was obtained by recursively removing nodes with a degree less than k. A residue domain wa defined as a set of residues belonging to a separate 2‐core. This means any residue within the domain had at least two contacts. The size of a domain was denoted as the number of residues contained in it. The residue domains with a size exceeding 4 were taken into account for analysis. The domain containing the maximum number of residues was referred to as the largest residue domain for each conformation. In order to further discuss the relationship between the residue domain and compact structure, consider a highly flexible protein PaaA2 (PDB ID: 3ZBE), was also considered which contained two stable α‐helices flanked by disordered regions. The PDB database provided 50 diverse conformations of PaaA2, and the domain involvement was calculated using these conformations (Figure S10, Supporting Information). Two regions, i.e., residues 15–28 and 41–57, exhibit high domain involvement. These regions were consistent with two α‐helix regions (residues 16–28 and 42–57) identified by NMR experiments.^[^ ^75^ ^]^

### The Details of the Correlation Function

The velocity–velocity correlation function reflected whether the residues move coherently. To estimate the velocity of the residue at frame t, the conformations of protein at neighboring frames t and t+1 were extracted from the trajectory (Δt between two neighboring frames is the time interval for saving coordinates, i.e., Δt = 100 ps). Subsequently, the two conformations were aligned to remove the translational and rotational motions of the whole protein, by minimizing the root‐mean‐square deviation (RMSD) of all atoms. After the alignment, the relative displacement of residue i can be calculated,

(6) $$\Delta {\vec{x}}_{i}={\vec{x}}_{i}\left(t+1\right)-{\vec{x}}_{i}\left(t\right)$$

where ${\vec{x}}_{i}\left(t\right)$ is the coordinate of residue i at frame t. The corresponding velocity of residue i can be estimated as

(7) $${\vec{u}}_{i}=\frac{\Delta {\vec{x}}_{i}}{\Delta t}$$

Then the correlation between the velocities of residues i‐j can be described as

(8) $$C_{ij}={\vec{u}}_{i}\cdot {\vec{u}}_{j}$$

With the consideration of correlations of all residue pairs throughout the conformation, the correlation function is defined as follows,

(9) $$C\left(r\right)=\frac{\sum_{i<j}^{N}\frac{C_{ij}}{\sqrt{C_{ii}C_{jj}}}\delta \left(r-r_{ij}\right)}{\sum_{i<j}^{N}\delta \left(r-r_{ij}\right)}$$

where δ(*r* − *r_ij_*) is the Dirac delta function and the mutual distance *r_ij_* of residue pairs, i‐j is computed as their center‐of‐mass distance.

### Protein Engineering

The genes for RGG‐rich motif of FUS and LAF‐1 were custom synthesized (GenScript, China) and the sequences were shown in Figure S1 (Supporting Information). Then the proteins were expressed for expression in *Escherichia Coli* (BL 21) cells and purified with Co^2+^ affinity chromatography using TALON resins (Takara, USA). Purified protein samples were dialyzed into deionized water and then lyophilized before use.

### Substrate Modification

Glass slides (24 × 40 mm^2^, VWR International, LLC) and glass coverslips (18 × 18 mm^2^, VWR International, LLC) were washed with acetone, deionized water, 1 m of KOH, and deionized water successively, and then dried under a steam of argon, followed by firing with propane to remove organic residues. After cooling to room temperature, the glass slides were immersed in methyl alcohol solution for use and the glass coverslips were soaked in methyl alcohol solution with 5% acetic acid for amino silanization at room temperature (R. T.) for 1h. Then, the coverslips were washed with methyl alcohol and dried under an argon flow. Next, the coverslips were immersed in bicarbonate buffer with Biotin‐PEG‐NHS (Nanocs, USA) and mPEG‐NHS (molar ration of 1:20) at pH 8.5 for 3h. Finally, the coverslips were washed with deionized water, dried under argon airflow, and stored in a vacuum for use.

### Protein Labeling

Cy3‐NHS (Cytiva, PA13101) and Alexa‐647‐Maleimide (Invitrogen, A20347) were used as donors and acceptors in the system. The proteins of interest were dissolved into 1× PBS with a molar concentration of 150 µm. Then, 200 µL protein solution was mixed with 50 µL of Dimethyl sulfoxide (DMSO) with Cy3 dye to incubate for 2h. The mixture was purified with a sephadex column chromatography to remove the excess dyes and then measured the protein concentration and labeling efficiency were with NanoDrop. Finally, the purified protein solution was concentrated to ≈200 µL and kept the concentration of 0.5–1.0 mM by adding trichloroethyl phosphate (TCEP) solution and incubation at least for 10 min before use. The labeling of Alexa‐647 following the same procedure as Cy3. The labeled proteins were diluted to 200 pm for use.

### Protein Modification

Adhering the coverslip to two sides of the glass slide with double sided tape and banding the other two sides with epoxide resin to build a cell allowing the sample entry and exit. The cell was washed with 200 µL PBS buffer three times and added 50 µl 0.2 mg mL^−1^ streptavidin (Invitrogen, S888) for incubation ≈2 min. Then the cell was washed three times with 200 µL PBS and incubated for 2 min with the mixture of 5 µm tris‐NTA‐Biotin solution (Biotechrabbit, BR1001201) and 12 µm NiCl_2_ solution. After washing with PBS three times to remove unreacted molecules. Next, 50 µL labeled protein was added into the sample cell for incubation in a dark place for 5 min. Finally, the cell was washed with PBS and filled with 50 µL imaging buffer. The imaging buffer contained 1 × PBS, 0.8% glucose (Sigma, D9559), 165 U mL^−1^ glucose oxidase (Sigma, G2133), 2170 U mL^−1^ catalase (Sigma, C40) and 2 mM Trolox (Sigma, 238813).

### Single‐Molecule Förster Resonance Energy Transfer (sm‐FRET) Experiments

The single‐molecule FRET experiments were performed with a total internal reflection fluorescence (TIRF) microscopy based on an Olympus IX‐71 microscopy and an oil immersion objective lens (UAPON 100 ×, N.A. = 1.49, Olympus) at R.T. Cy3 was excited by a 532‐nm laser (MLL‐S‐532‐B‐80 mW) and the emission fluorescence of Cy3 and that of Alexa‐647 was split into two channels by a dichroic filter (FF640‐FDi01, Semrock). The emission fluorescence of two channels passed through two band‐pass filters (FF01‐585/40 and FF01‐675‐67, Semrock), respectively, and the final fluorescence signals were collected by an electron‐multi‐plying charge‐coupled device camera (IXon897, Andor Technology).

### Statistical Analysis

For each FRET trajectory, the transfer efficiency was converted to the distance between the dye pair, and the power spectrum of distance was calculated using the equations mentioned above. A total of 62 trajectories for FUS‐C and 40 trajectories for LAF‐N were used for analysis. The experimental power spectrum for each IDP was the average of all independent FRET trajectories and was expressed as mean values ± SD. All analyses were conducted in the Python 3.9 environment.

### Data and Code Availability

All data needed to evaluate the conclusions in the paper are present in the paper and/or the Supplementary Materials. Additional data related to this paper are available from the corresponding authors upon request. The Gromacs input files used for simulations are freely available from GitHub (https://github.com/Song‐1999/simulation_files).

## Conflict of Interest

The authors declare no conflict of interest.

## Author Contributions

H.S. and J.L. conceptualized and designed the project. H.S. performed all simulations and conducted the data analysis. J.C., H.L., H.S. and J.L. designed the experiments. J.C. performed the experiments. H.S., J.L. and H.L. wrote the manuscript. All authors discussed and revised the manuscript.

## Supporting information



Supporting Information

## Data Availability

The data that support the findings of this study are available from the corresponding author upon reasonable request.
