# Supplementary material for: Scale‐free Spatio‐temporal Correlations in Conformational Fluctuations of Intrinsically Disordered Proteins
Source: Adv Sci (Weinh). 2025 Jan 14;12(9):2412989. doi: 10.1002/advs.202412989 (PMC11884614; doi:10.1002/advs.202412989)
Supplement: Supplementary file 1 — Supporting Information [file ADVS-12-2412989-s001.docx]

Supplementary Information for

**Scale-free Spatio-temporal Correlations in Conformational Fluctuations of Intrinsically Disordered Proteins**

Haoyu Song^1^, Jian Cui^2^, Guorong Hu^1^, Long Xiong^3^, Yanee Wutthinitikornkit^1^, Hai Lei^1, *^ and Jingyuan Li^1, *^

^1^School of Physics, Zhejiang University, Hangzhou 310058, PR China

^2^Collaborative Innovation Center of Advanced Microstructures, National Laboratory of Solid State Microstructure, Department of Physics, Nanjing University, Nanjing, 210093, PR China

^3^School of Physics and Astronomy, Yunnan University, Kunming 650091, PR China

*Corresponding Author: [leihai@zju.edu.cn](mailto:leihai@zju.edu.cn) (H. Lei); [jingyuanli@zju.edu.cn](mailto:jingyuanli@zju.edu.cn) (J. Li)

**Figure S1.** The sequences of IDPs studied in this work. (a) FUS-C (residues 454-526 of FUS), (b) LAF-N (residues 1-168 of LAF-1) and (c) TAF-C (residues 386-592 of TAF15). (d) The sequence of FUS-C used for smFRET. The endogenous residue K510 is labeled with donor Cy3. An additional Cys is introduced at the N-terminus of FUS-C (position 453) and is labeled with acceptor Alexa 647. (e) Another sequence of FUS-C to examine the influence of labelling position. Two additional Cys are introduced at its both termini. (f) The sequence of LAF-N used for smFRET. Additional Cys and Lys are introduced at its two termini.


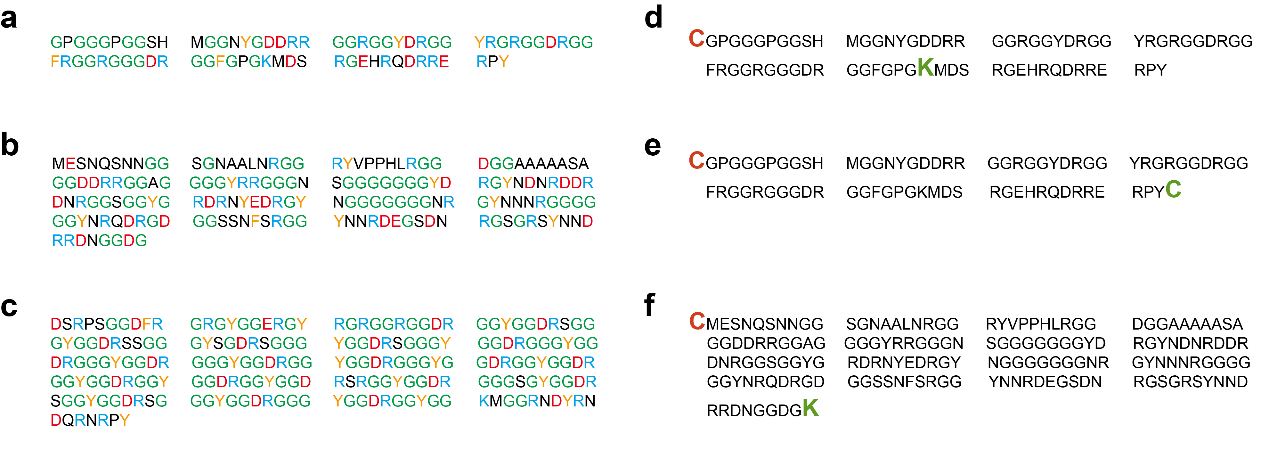


**Figure S2.** The conformational ensembles of three IDPs based on ten independent 1-μs simulations. The distributions of $R_{ee}$ for (a) FUS-C, (b) LAF-N and (c) TAF-C, and the distributions of $R_{g}$ (d-f) are shown. The average values and the standard deviations are listed in the figures. All these IDPs exhibit large-scale conformational fluctuations.


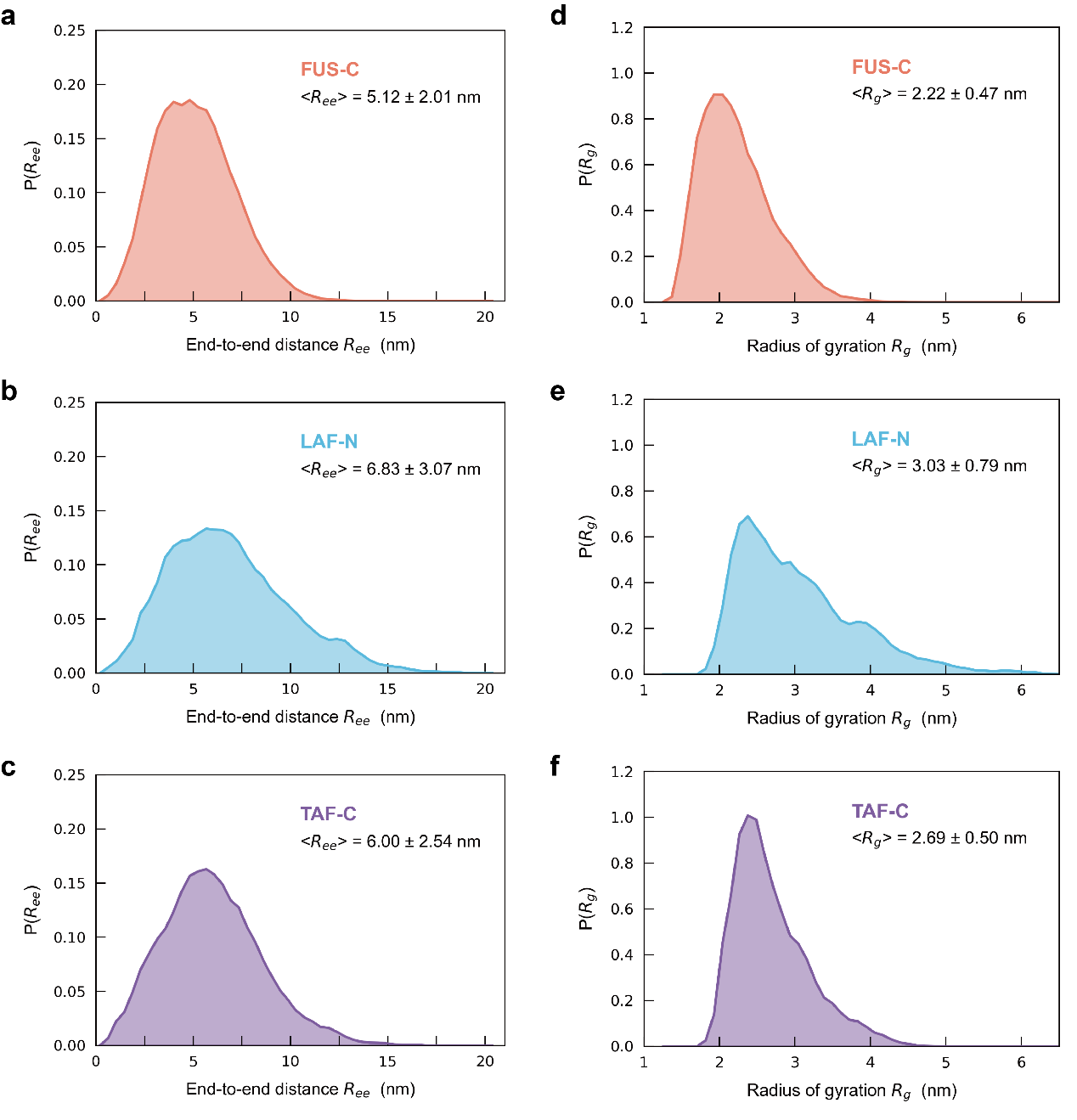


**Figure S3.** The power spectrum with error bar of three IDPs (a) FUS-C, (b) LAF-N and (c) TAF-C. The error bar (mean ± s.d.) is calculated based on all independent MD trajectories and is represented by the gray-shaded area.


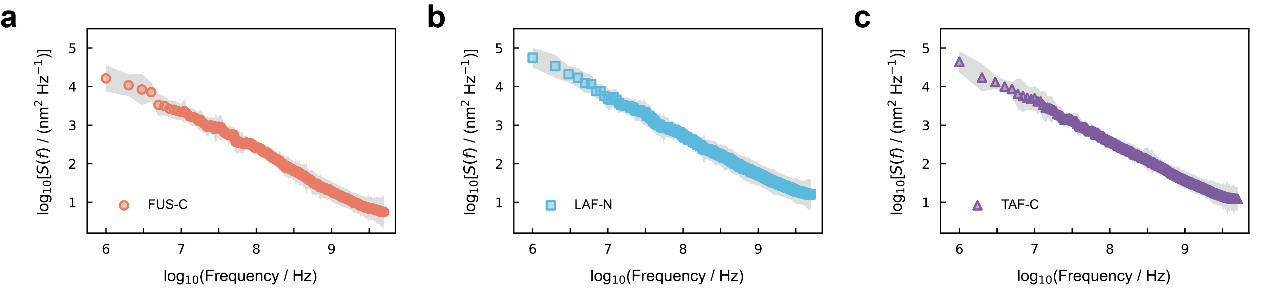


**Figure S4.** Schematic diagram of polymer sequences. The sequences are composed of hydrophobic beads (red dots) and polar beads (blue dots). In each sequence, the positions of two types of beads are settled randomly and the proportion of hydrophobic beads is around 0.5. A total of 100 independent sequences are constructed for the simulations, of which 10 sequences are shown here.


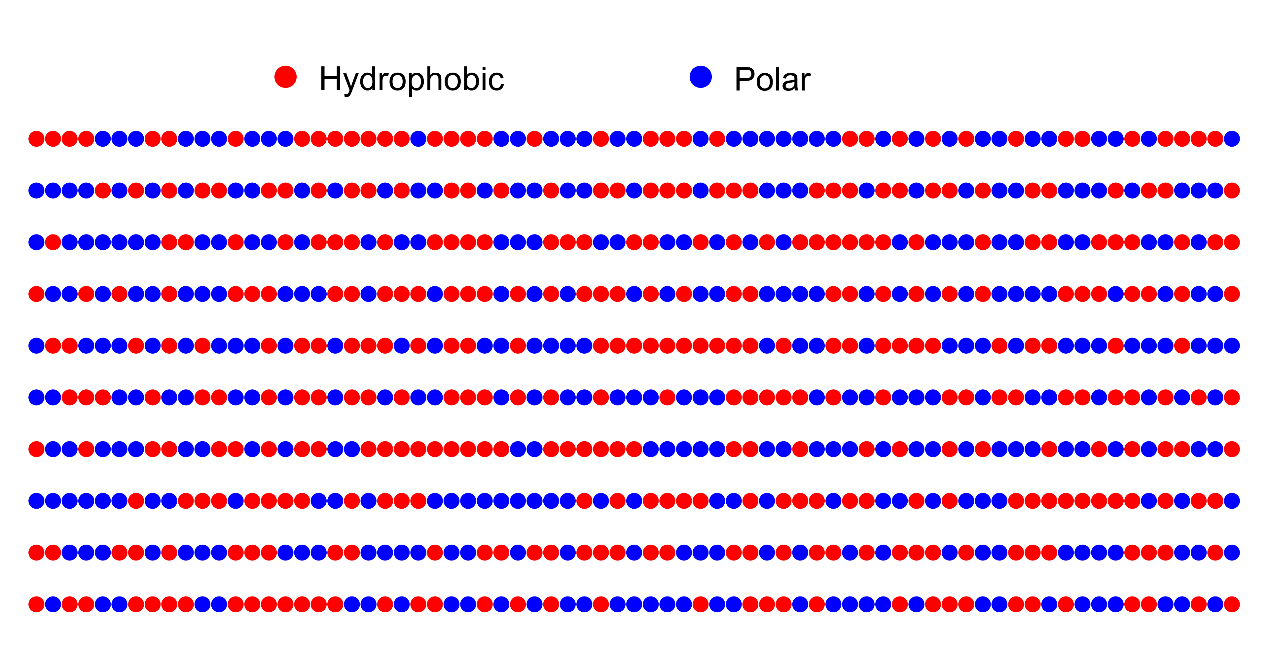


**Figure S5.** The absence of scale-free temporal and spatial correlations in polymer system. (a) The $R_{ee}$ distribution of polymers based on all 100 trajectories. Insert, the scaling of average internal distances $R_{ij}$ with sequence separation $\left| i-j \right|$, and its slope can be regarded as Flory exponent. Both the distribution and Flory exponent are similar to the distribution of IDP. (b) The $R_{ee}$ fluctuation of one representative trajectory. The polymer also undergoes large-scale conformational fluctuation as IDP. The power spectrum (c) and domain size distribution (d) of the representative trajectory shown in (b). (e) The power spectrum of polymers based on all trajectories. It follows $1/f$ noise in high-frequency region (colored in blue), but deviates from $1/f$ behavior in the low-frequency region (colored in yellow). The exponent $\beta$ in low-frequency region is merely ~0.54, which is biased towards white noise. (f) The domain size distribution of polymers based on all trajectories. In double-logarithmic plot, its distribution does not present as a straight line. There is no region in the distribution exhibiting a constant slope. With the increasing of domain size, the probability distribution rapidly decreases, deviating from the power law. On the account of both power spectrum and domain distribution, polymers do not exhibit scale-free spatio-temporal correlations.


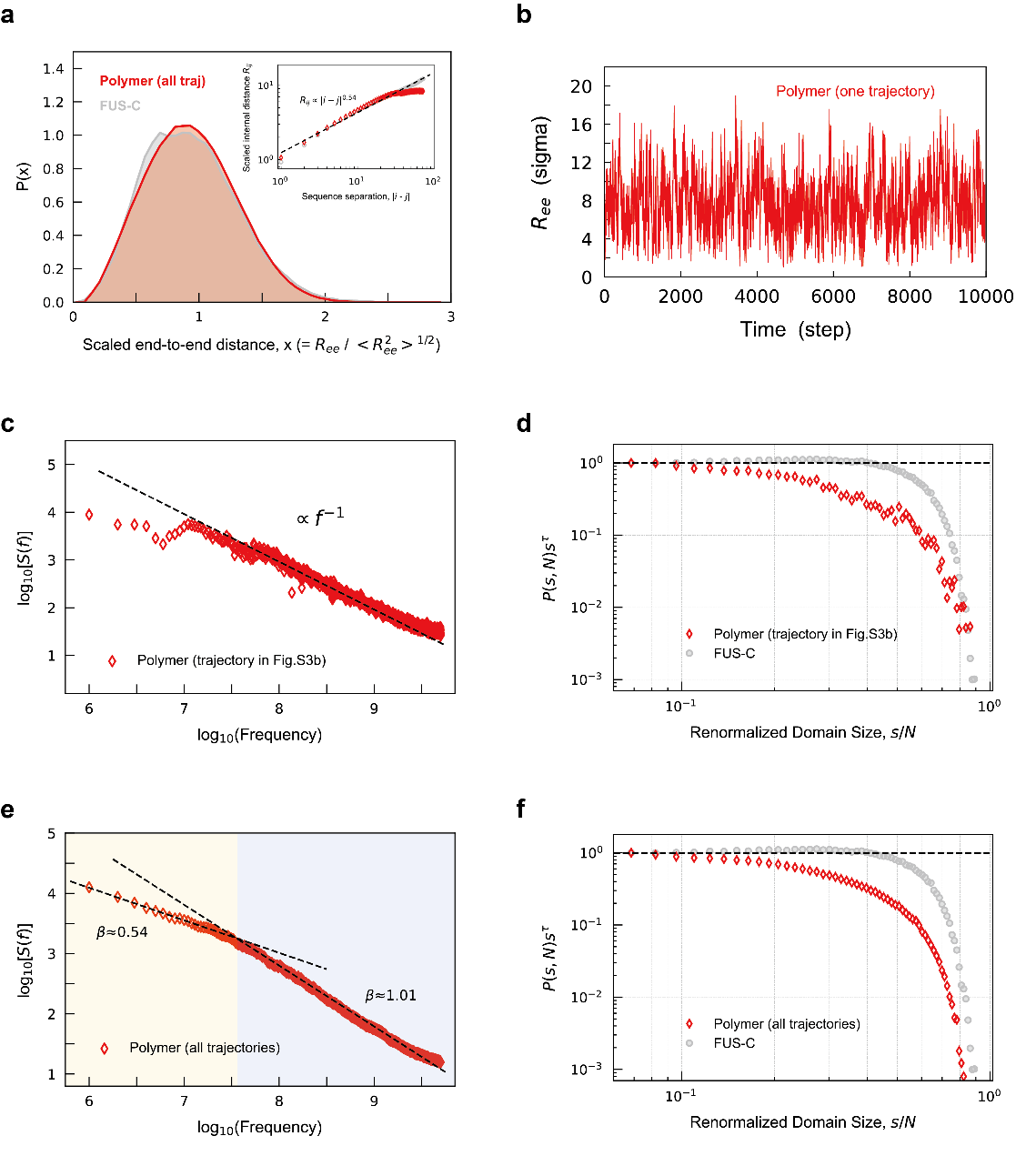


**Figure S6.** The conformation of FUS-C in 500 mM NaCl solution. Under high salt concentration, the scale-free spatio-temporal correlations are absent, and the structural heterogeneity is reduced. (a) The $R_{ee}$ distributions of FUS-C in 500 mM NaCl with respect to physiological concentration (150 mM NaCl). The global conformation in 500 mM becomes more compact. (b) The domain size distribution and (c) the power spectrum of FUS-C in 500 mM NaCl. The domain distribution significantly deviates from power law. And the power spectrum exhibits a tendency towards Brownian noise as the exponent $\beta$ increases to ~1.3. (d) The domain involvement of the residues along the sequence of FUS-C in 150 mM and 500 mM NaCl. The mean values of involvement in domain-prone region and C-terminal region (gray-shaded areas) are 0.41 and 0.19 for 150 mM condition. These values increase to 0.71 and 0.60 for 500 mM condition, respectively. The difference between two regions is reduced at high-salt condition.


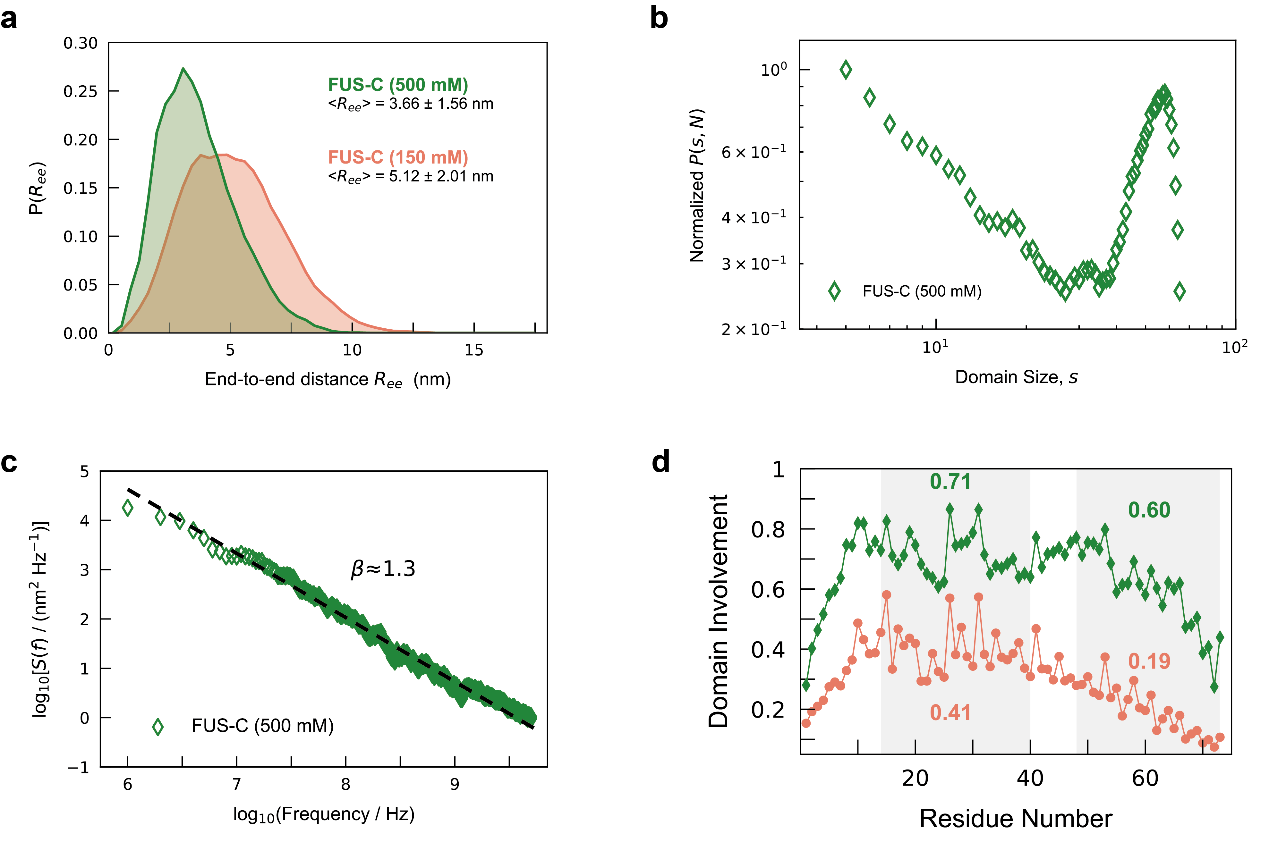


**Figure S7.** The evolution of the distance between G454 and M511 of FUS-C. To better compare with the experiments which measure the FRET efficiency between an additional C453 and K510 (shown in Fig. S1d), the distance between G454 and M511 are calculated. (a) The distribution of distance between G454 and M511. The distance is converted to the FRET efficiency and the mean FRET efficiency based on simulation is estimated as $\left\langle E \right\rangle_{simulation}$ ~0.67, close to the experimental result $\left\langle E \right\rangle$ ~0.63. (b) The power spectrum of the evolution of distance G454-M511. It has similar $1/f$ behavior as the entire FUS-C.


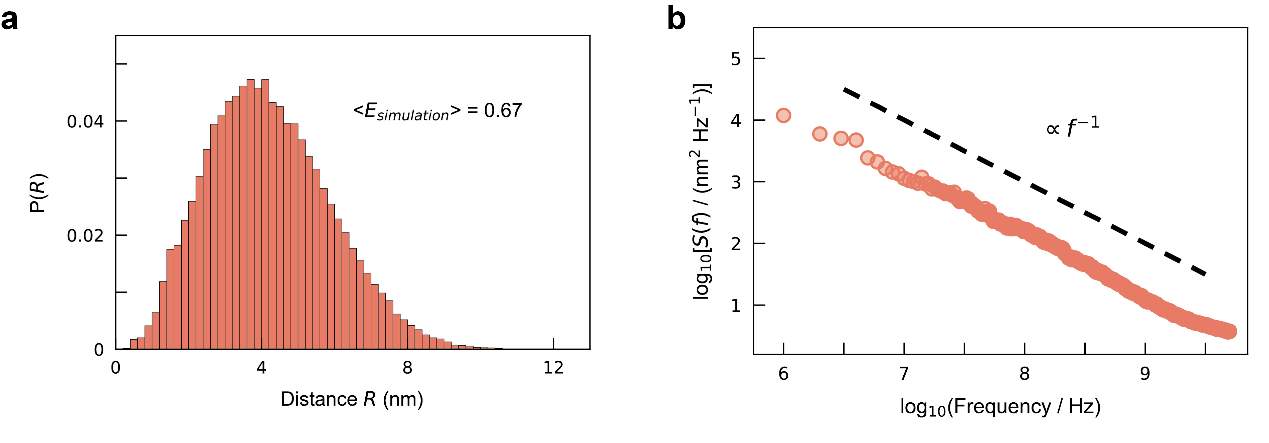


**Figure S8.** The smFRET experimental results of FUS-C labeled at its both termini. (a) The donor and acceptor fluorescence intensities of the representative trajectory. (b) The FRET efficiency trajectory. (c) The histogram of FRET efficiency. The mean transfer efficiency of the experiment is $\left\langle E \right\rangle$ ~0.51 (black dashed lines). The mean value based on simulations $\left\langle E \right\rangle_{simulation}$ ~0.52 is consistent with the experimental results. (d) The power spectrum of the evolution of the end-to-end distance (after conversion from FRET efficiency). It follows the form of power-law with an exponent $\beta=0.94$, also indicating the $1/f$ behavior. The grey area is error bar (mean ± s.d.) based on 21 trajectories.


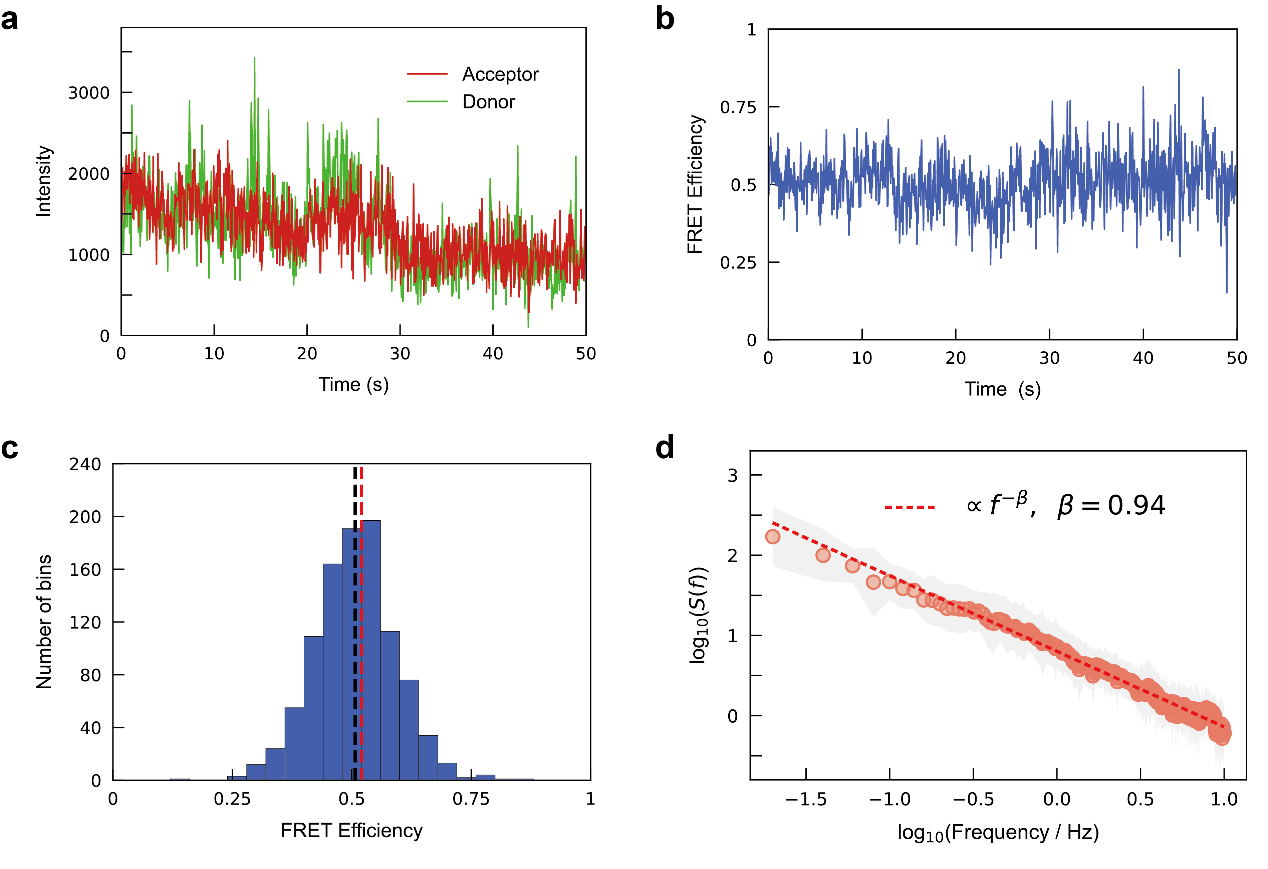


**Figure S9.** The power spectrum of end-to-end distance of LAF-N within condensate (containing 40 LAF-N motifs). The LAF-N within condensate can still exhibit 1/f power spectrum.


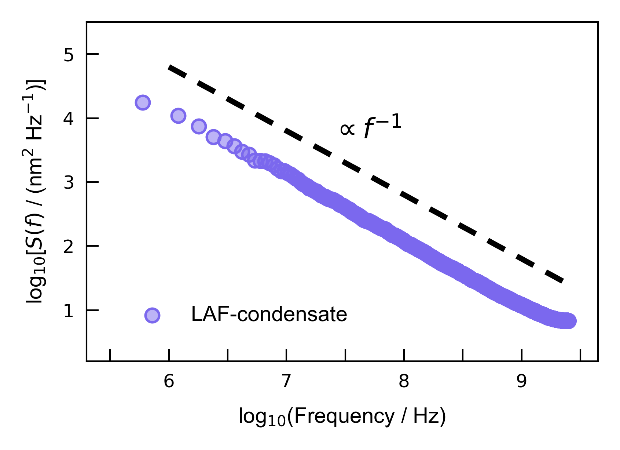


**Figure S10.** (a) Four conformations of PaaA2. PaaA2 contains two stable α-helices flanked by disordered regions, and exhibits substantial conformation changes. (b) The domain involvement of residues along PaaA2 sequence. Two regions (residues 15-28 and 41-57; gray-shaded areas) exhibit high domain involvement. These regions are consistent with two α-helix regions identified by NMR experiment.


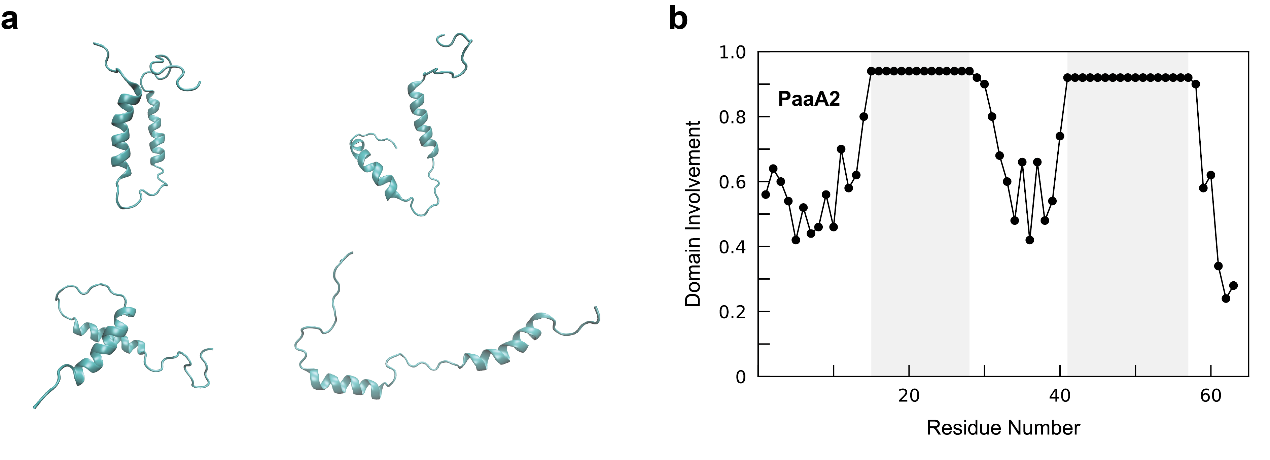


**Table S1.** The difference of residue compositions between domain-prone region and entire sequence. Three types of residues, i.e., Gly, Arg and Tyr, are enriched in domain-prone regions in all three proteins.

|  | **FUS-C** | **LAF-N** | **TAF-C** |
| --- | --- | --- | --- |
| **Gly (G)** | 2.9% | 5.9% | 5.8% |
| **Arg (R)** | 7.4% | 1.8% | 0.5% |
| **Tyr (Y)** | 6.5% | 2.4% | 1.4% |
